# Biochemical and Functional Characterization of the Interaction between Liprin-α1 and GIT1: Implications for the Regulation of Cell Motility

**DOI:** 10.1371/journal.pone.0020757

**Published:** 2011-06-13

**Authors:** Claudia Asperti, Veronica Astro, Emanuela Pettinato, Simona Paris, Angela Bachi, Ivan de Curtis

**Affiliations:** 1 Division of Neuroscience, San Raffaele Scientific Institute and San Raffaele University, Milano, Italy; 2 Division of Genetics and Cell Biology, San Raffaele Scientific Institute, Milano, Italy; Kings College London, United Kingdom

## Abstract

We have previously identified the scaffold protein liprin-α1 as an important regulator of integrin-mediated cell motility and tumor cell invasion. Liprin-α1 may interact with different proteins, and the functional significance of these interactions in the regulation of cell motility is poorly known. Here we have addressed the involvement of the liprin-α1 partner GIT1 in liprin-α1-mediated effects on cell spreading and migration. GIT1 depletion inhibited spreading by affecting the lamellipodia, and prevented liprin-α1-enhanced spreading. Conversely inhibition of the formation of the liprin-α1-GIT complex by expression of liprin-ΔCC3 could still enhance spreading, although to a lesser extent compared to full length liprin-α1. No cumulative effects were observed after depletion of both liprin-α1 and GIT1, suggesting that the two proteins belong to the same signaling network in the regulation of cell spreading. Our data suggest that liprin-α1 may compete with paxillin for binding to GIT1, while binding of βPIX to GIT1 was unaffected by the presence of liprin-α1. Interestingly, GIT and liprin-α1 reciprocally regulated their subcellular localization, since liprin-α1 overexpression, but not the GIT binding-defective liprin-ΔCC3 mutant, affected the localization of endogenous GIT at peripheral and mature central focal adhesions, while the expression of a truncated, active form of GIT1 enhanced the localization of endogenous liprin-α1 at the edge of spreading cells. Moreover, GIT1 was required for liprin-α1-enhanced haptotatic migration, although the direct interaction between liprin-α1 and GIT1 was not needed. Our findings show that the functional interaction between liprin-α1 and GIT1 cooperate in the regulation of integrin-dependent cell spreading and motility on extracellular matrix. These findings and the possible competition of liprin-α1 with paxillin for binding to GIT1 suggest that alternative binding of GIT1 to either liprin-α1 or paxillin plays distinct roles in different phases of the protrusive activity in the cell.

## Introduction

Cell migration requires complex molecular events that need to be finely regulated in time and space [1]. GIT1 (G protein-coupled receptor kinase-interacting protein 1) and GIT2/PKL form a family of multi-domain ArfGAP proteins with scaffolding activity, which are implicated in the regulation of cell adhesion and migration on extracellular matrix [2]. They interact via an SHD (Spa2 homology domain) with the components of the PIX (p21-activated kinase-interacting exchange factor) family of guanine nucleotide exchanging factors for Rac and Cdc42 GTPases [3]–[5]. Moreover, the carboxy-terminal region of GIT proteins can interact with the adaptor proteins paxillin [6], [7] and liprin-α1 [8], both implicated in the formation and turnover of integrin-mediated FAs (focal adhesions) [9]–[11].

GIT proteins are involved in different pathways that regulate cell motility. For example, GIT1 is involved in EGF-dependent vascular smooth muscle cell migration [12], while the second member of the family, GIT2 is a key player for chemotactic directionality in stimulated neutrophils [13], and is required for PDGF-dependent directional cell migration and cell polarity, but not for random migration [14].

It has been proposed that GIT1 may cycle between at least three distinct subcellular compartments, including FAs, leading edge, and cytoplasmic compartments, and the functional interaction between GIT1, βPIX and PAK has been associated to cell protrusive activity and migration [15], [16]. On the other hand, the precise function of the GIT complexes in cell motility is still insufficiently understood, and existing findings have led to conflicting reports on whether the recruitment of GIT-mediated complexes positively [17] or negatively [18] affect Rac-mediated protrusion.

The localization of GIT1 at the leading edge may play a role in recruiting the GTPase activator βPIX and the Rac effector PAK at the same location, thus restricting the activity of Rac1 to the front of motile cells where actin assembly is needed [19]–[21]. It has been shown that GIT1 regulates the protrusive activity at the cell border, and that the GIT1/PIX/PAK complex is recruited by the FA protein paxillin at dynamic peripheral adhesive structures to regulate their turnover [17].

Liprins are a family of scaffold proteins that include the liprin-α and -β subfamilies [10]. Liprin-α proteins are multi-domain proteins that can interact directly with several binding partners. Recent work has revealed that liprin-α1 is an essential regulator of cell motility and tumor cell invasion [11], [22]–[24] but the exact implication and role of the different liprin-α/partner complexes in the regulation of cell motility are poorly understood [25]. We have shown that the interaction of GIT1 with liprin-α1 and paxillin must be regulated. In fact, both liprin-α1 and paxillin interact poorly with the full length GIT1 protein, while they interact efficiently with carboxy-terminal fragments of GIT1 or with GIT1 polypeptides with limited internal deletions [26], suggesting that GIT1 function is regulated by an intramolecular mechanism. Accordingly, overexpression of the “active” truncated GIT1-C protein, but not the full length protein, leads to enhanced cell spreading [26].

In this study we have analyzed the biochemical and functional interaction between liprin-α1 and GIT1 to explore the role of this interaction in cell motility. By co-immunoprecipitation experiments we have shown that GIT1 may form alternative complexes with either paxillin or liprin-α1. Moreover, we found that GIT1 is required for liprin-α1-mediated cell spreading and migration, although the direct interaction between the two proteins does not appear to be essential for these processes. Finally, we demonstrated a reciprocal effect of liprin and GIT on their localization at FAs at the cell edge, which correlated with the ability of the two proteins to interact with each other.

## Results and Discussion

### Liprin-α1 interferes with the binding of paxillin, but not of βPIX to GIT1

Regions of interaction between liprin-α1 and GIT1 have been previously identified by a yeast two-hybrid assay. A central fragment of liprin-α1 (amino acid residues 603–673) interacted with the carboxy-terminal region of GIT1, and the interaction between the two full-length proteins was confirmed by co-immunoprecipitation from lysates from HEK-293T cells or from the synaptosomal fraction of adult rat brain [8], [27]. By further investigating the interaction of GIT1 with liprin-α1, we confirmed the interaction of liprin-α with the carboxy-terminal part of GIT1 by pull down from embryonic chick brain lysates with the ZZ-GIT1-C2 fusion protein pre-bound to IgG-Sepharose. A specific band of about 160 kDa was eluted from IgG beads coupled to ZZ-GIT1-C2 compared to control IgG beads (Fig. S1, A). Mass spectroscopy analysis of this band revealed several peptides corresponding to peptide sequences of the human liprin-α2 protein (Fig. S1, B), a member of the liprin-α family prevalently expressed in neural tissue. This finding confirms previous results on the identification of the interaction of liprin-α proteins with GIT1 [8]. Here, for the following functional and biochemical analysis we have then switched to consider the ubiquitously expressed liprin-α1 protein. Since overexpressed liprin-α1 interacts poorly with overexpressed full length GIT1, but efficiently with the GIT1-C2 carboxy-terminal polypeptide [26], we further investigated the requirements for the interaction between the two proteins by co-transfecting COS7 cells with one of several GIT1 truncation mutants together with either full length liprin-α1 or with the Myc-tagged liprin-F3 fragment (amino acid residues 347–675 of human liprin-α1) that includes the GIT1-binding region (Fig. S1, G–H). An extended carboxy-terminal fragment was required for reproducible and efficient co-immunoprecipitation of the two proteins from cell lysates (Fig. S1, C,E–F). Carboxy-terminal fragments shorter than GIT1-C gave weak or no interaction with liprin-α1. In particular, immunoprecipitation of Myc-liprin-F3 from co-transfected cells showed no interaction between the liprin-α1 fragment and FLAG-GIT1(512–740) (Fig. S1, E). The interaction was absent also in reciprocal immunoprecipitations using anti-FLAG antibodies (data not shown).

Paxillin interacts with the carboxy-terminal region of rat GIT1 including residues 640–770 (residues 610–740 in chick GIT1) via the LD motifs [7], [28]. As expected, endogenous paxillin co-precipitated with FLAG-GIT1(512–740) that includes the full paxillin binding region [7] (Fig. S1, E). On the other hand, constructs including short deletions at the carboxy-terminus [FLAG-GIT1(229–680) and FLAG-GIT1(229–667)] abolished the interaction with both liprin-α1 and paxillin (Fig. S1, C–D). Altogether the results show that an extended region of the carboxy-terminal portion of GIT1 is required for efficient interaction with liprin-α1, and that the region of GIT1 required for the binding to liprin-α1 includes the paxillin-binding region.

**Figure 1:**
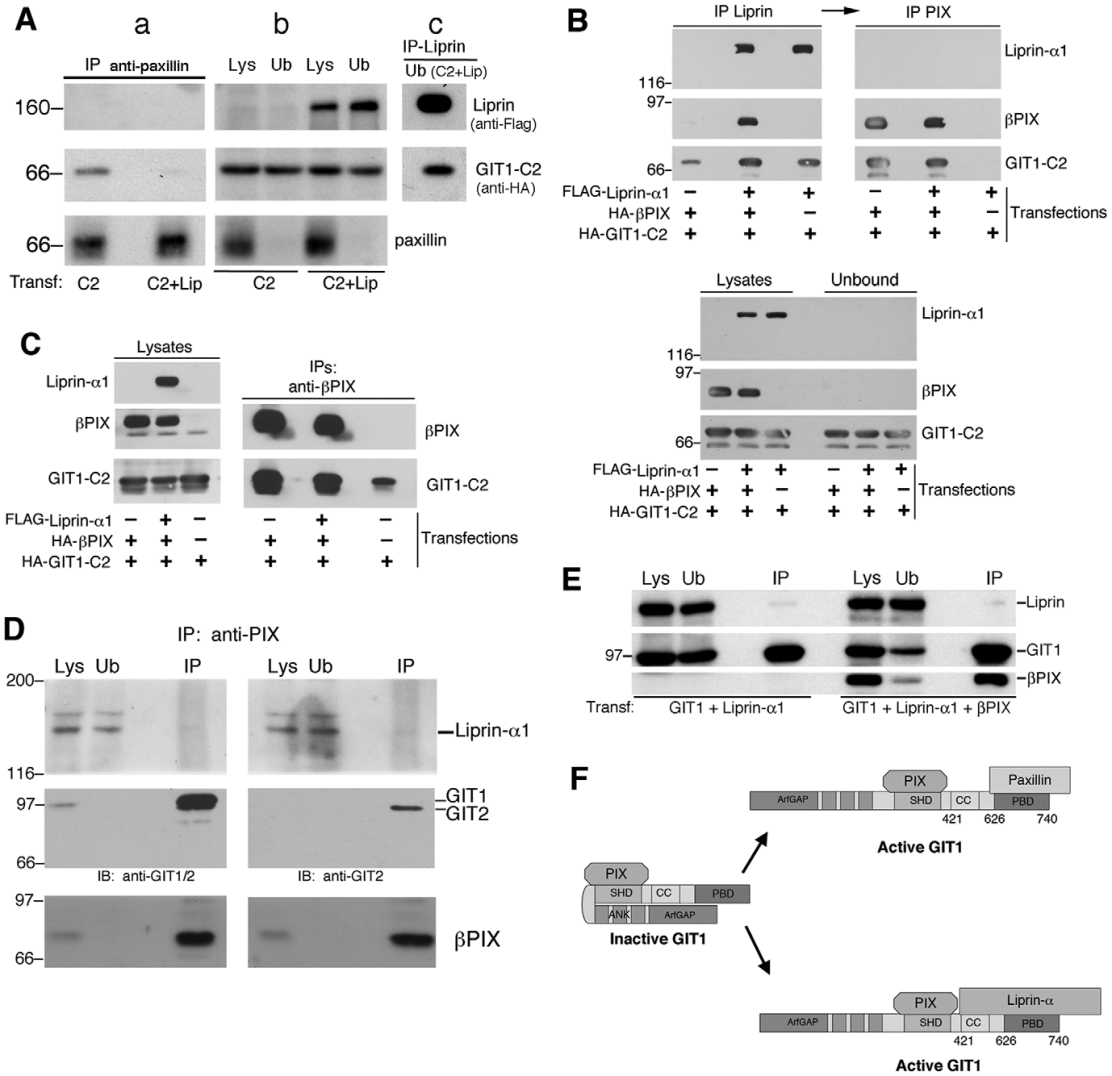
Binding of liprin-α1 to GIT1-C2 prevents binding of paxillin to GIT1-C2. (A) Lysates were prepared from COS7 cells transfected with either HA-GIT1-C2 (C2) or co-transfected with HA-GIT1-C2 and FLAG-liprin-α1 (C2+Lip). Aliquots of the lysates were used for immunoprecipitation with anti-paxillin antibodies (IP anti-paxillin, 400 µg of protein per IP). Filters with immunoprecipitates (a), and with 100 µg of both lysates (Lys) and unbound fractions after IP (Ub) (b) were cut and immunoblotted with anti-Flag to detect Flag-liprin-α1 (upper filters, only one of the duplicated immunoprecipitations is shown); since GIT1-C2 and paxillin migrate at similar positions on gels, the lower parts of the filters from the duplicated immunoprecipitations were used as follows: one set of filters (a+b) was incubated with anti-HA to detect HA-GIT1-C2 (middle blots), and one set was incubated with anti-paxillin to detect endogenous paxillin (lower blots). Paxillin was absent from the unbound fractions after immunoprecipitation (Ub). (c) The unbound fraction (300 µg) after immunoprecipitation with anti-paxillin from the lysate of cells co-transfected with HA-GIT1-C2 and FLAG-liprin-α1 [Ub(C2+Lip)], was re-immunoprecipitated with anti-liprin antibody, to reveal the presence of the liprin-α1/GIT1-C2 complex in the lysate. (B) Binding of liprin-α1 to GIT1-C2 does not prevent binding of βPIX to GIT1-C2. Identification of a ternary complex among liprin-α1, βPIX and GIT1-C2. COS7 cells co-transfected to express the indicated combinations of HA-GIT1-C2, HA-βPIX, and FLAG-liprin-α1 were immunoprecipitated with anti-FLAG antibodies (top blots on the left). Aliquots of the unbound fraction after the first round of immunoprecipitations were re-immunoprecipitated with anti-βPIX antibodies (top blots on the right). Filters including immunoprecipitations (IP), lysates (Lys), and unbound fractions after the second round of immunoprecipitations (Ub) were cut and blotted as indicated (lower blots). (C) Liprin-α1 does not interfere with the interaction of βPIX with GIT-C2. COS7 cells co-transfected to express the indicated combinations of HA-GIT1-C2, HA-βPIX, and FLAG-liprin-α1 were immunoprecipitated with anti-βPIX antibodies. Filters including aliquots of lysates and the immunoprecipitations (IP) were cut and blotted as indicated. (D) A COS7 cell lysate (1 mg protein) was immunoprecipitated with anti-βPIX antibodies. Immunoprecipitate (IP) and equal amounts (100 µg) of lysate (Lys) and unbound fraction (Ub) were blotted with anti-GIT (mAb PKL, recognizing both GIT1 and GIT2 proteins, on the left; or anti-GIT2-specific pAb, on the right), βPIX, or anti-liprin-α1 antibodies. Blot with anti-GIT antibody was performed after stripping the filter incubated for βPIX. (E) binding of βPIX to full length GIT1 does not enhance the binding of liprin-α1 to GIT1. COS7 cells were co-transfected with FLAG-liprin-α1 and FLAG-GIT1, or with FLAG-liprin-α1 and FLAG-GIT1 and HA-βPIX. 200 µg of each lysate were immunoprecipitated with anti-GIT1 antiserum. Lysates (Lys, 50 µg), unbound fractions (Ub, 50 µg) and immunoprecipitates were blotted and incubated with antibodies specific for the indicated proteins. Overexpression of βPix did not increase the interaction of liprin-α1 with GIT1. (F) Model for the regulated interaction of GIT1 with paxillin and liprin-α1. Either ligand binds poorly to full length GIT1. We hypothesize that activation of GIT1 by so far unknown mechanisms is required for the formation of either GIT1/paxillin or GIT1/liprin-α1 complexes.

Based on these findings, we tested the hypothesis that liprin-α1 may interfere with the binding of paxillin to the carboxy-terminus of GIT1 in the cell. For this, we first immunoprecipitated endogenous paxillin from lysates of cells transfected either with HA-GIT1-C2 alone, or with both HA-GIT1-C2 and full length FLAG-liprin-α1. Under conditions in which endogenous paxillin was virtually immunodepleted from lysates (Fig. 1, A, panels a and b), the interaction of paxillin with HA-GIT1-C2 was strongly reduced in the lysates from co-transfected cells (Fig. 1, A, panel a). We then tested the hypothesis that the decrease of binding of paxillin to GIT1-C2 may be due to binding of the overexpressed liprin-α1 to GIT1-C2 itself. For this, the unbound fraction after immunoprecipitation with anti-paxillin from lysates of cells co-transfected with HA-GIT1-C2 and FLAG-liprin-α1, was used in a second round of immunoprecipitation with anti-liprin-α1 antibody (Fig. 1, A, panel c). This immunoprecipitation showed a strong interaction of FLAG-liprin-α1 with HA-GIT1-C2 (Fig. 1, A, panel c). These data suggest that binding of overexpressed liprin-α1 to the carboxy-terminal portion of GIT1 interferes with the binding of paxillin to the same region of GIT1, and indicate that the formation of a trimeric liprin-α1/GIT1/paxillin complex in the cell is not likely.

GIT1 and βPIX form stable hetero-complexes in COS7 cells [26]. We thus tested if βPIX binding to the SHD domain of GIT1 interfered with the binding of liprin-α1 to the contiguous GIT1 carboxy-terminus. We used co-immunoprecipitation from transfected cell lysates to test for the possible interference between liprin-α1 and βPIX binding to GIT1. COS7 cells co-transfected with HA-GIT1-C2 and HA-βPIX, with HA-GIT1-C2 and FLAG-Liprin-α1, or triple-transfected with HA-GIT1-C2, HA-βPIX and FLAG-Liprin-α1 were immunoprecipitated with anti-FLAG antibodies. Similar amounts of GIT1-C2 were co-immunoprecipitated with anti-liprin-α1 antibodies in the presence or absence of βPIX, indicating that binding of liprin-α1 to GIT1-C2 did not affect the interaction of GIT1-C2 with βPIX (Fig. 1, B). These results indicate that GIT1 may be found in complex with both βPIX and liprin-α1 at the same time. On the other side, we found that immunoprecipitation of βPIX from co-transfected cells resulted in efficient co-precipitation of GIT1-C2 both in the presence and absence of liprin-α1 (Fig. 1, C). These results show that a trimeric βPIX/GIT1/Liprin-α1 complex may form in the cell.

We have previously shown that binding of paxillin to endogenous or overexpressed GIT1/βPIX complexes is usually undetectable and requires GIT1 activation by unknown mechanisms. Likewise, liprin-α1 interacts poorly with full length GIT1, while interacts efficiently with GIT1 deletion mutants that mimic an activated form of GIT1 [26]. As for paxillin, we could not detect the interaction of endogenous liprin-α1 with the endogenous GIT/PIX complexes after immunoprecipitation from COS7 lysates (Fig. 1, D). Moreover, as already shown for paxillin, co-expression of βPIX did not improve the association of overexpressed liprin-α1 to overexpressed full length GIT1 (Fig. 1, E). Therefore, we can conclude that binding of βPIX to GIT1 is not sufficient to activate the binding of these ligands to the carboxy-terminal portion of GIT1.

We have previously hypothesized that activation of GIT1 by so far unknown mechanisms is required for the formation of either GIT1/paxillin or GIT1/liprin-α1 complexes [26]. Altogether, the biochemical analysis described here indicates that the region of contact between liprin-α1 and GIT1 involves the carboxy-terminal half of the GIT1 polypeptide. These data also confirm the hypothesis that βPIX may represent a stable partner of GIT1, while GIT1 may change its carboxy-terminal partners according to the cell's requirements (Fig. 1, F). This model is also supported by our previous data indicating that in contrast to endogenous paxillin, most if not all endogenous βPIX is found in complex with endogenous GIT1 proteins in COS7 cells [26]. The mechanisms for the proposed intramolecular switch are unknown. Since our published work indicates the association of the aminoterminal portion of GIT1 to the carboxyterminal part of the protein, one possibility is that the ArfGAP domain is not only structurally, but also functionally relevant for the activation of GIT1. It is also worth noting that in lysates from cells overexpressing GIT1 minor specific bands of lower molecular weight are detectable (Fig. S1, C). Although we noticed that the abundance of these fragments may vary in different experiments, one can not rule out at this point that an alternative way to activate GIT1 may derive from the proteolytic cleavage of the full length protein to produce one or more types of active carboxyterminal fragments, which could be able to bind either paxillin or liprin.

### GIT1 is required for efficient liprin-α1-mediated cell spreading

**Figure 2:**
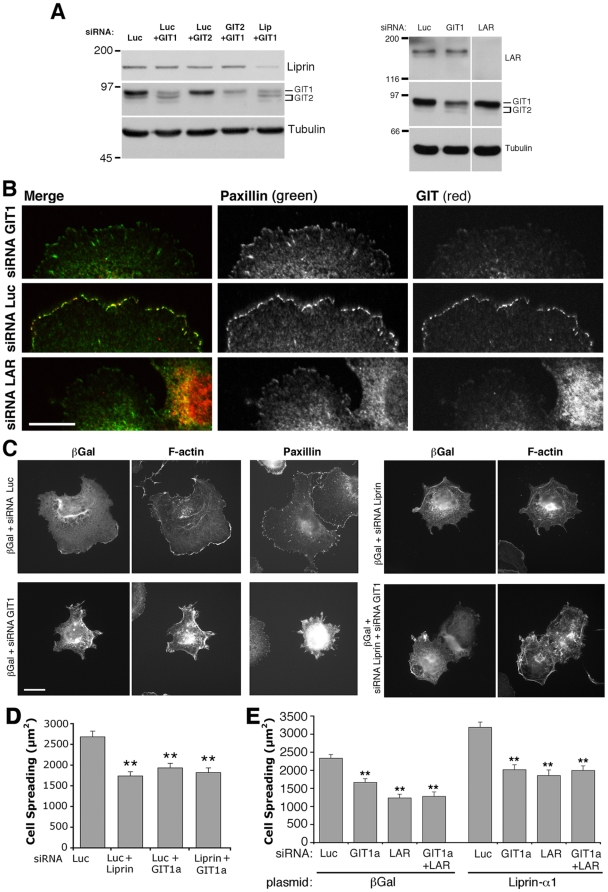
GIT1 and LAR depletion inhibit cell spreading and prevent enhanced spreading by liprin-α1 overexpression. (A) Specific and control (Luc = luciferase) siRNA duplexes were used to downregulate the expression of endogenous GIT1, GIT2, liprin-α1 and LAR in COS7 cells. Cells were lysed 2 days after transfection with siRNAs. After SDS-PAGE and blotting of 50 µg of each lysate, filters were incubated with antibodies for the indicated proteins. For each specific siRNA, we could only detect the downregulation of the specific target proteins with respect to the other endogenous proteins tested as controls. For GIT1 and GIT2, a monoclonal antibody recognizing both proteins was used here. (B) The signal for endogenous GIT (red) is strongly decreased at paxillin-positive (green) focal adhesions following transfection with siRNA for either GIT1 (top) or LAR (bottom) when compared to control cells (middle). Scale bar, 5 µm. (C) COS7 cells were trypsinized 2 days after co-transfection with the indicated siRNAs and βgalactosidase (βGal), and plated 1 h on FN before immunostaining. Scale bar, 20 µm. (D, E) Quantification of spreading after replating 1 h on FN of cells co-transfected for 2 days with siRNAs (D: means ±SEM; n = 100 cells per condition), or with siRNAs and plasmids for either βgalactosidase or liprin-α1 (E: means ±SEM, n = 80–90 cells per condition from 2 experiments). **P<0.01.

Liprin-α1 is a regulator of cell motility required for the efficient integrin-mediated spreading of COS7 cells [11]. COS7 cells express mainly GIT1, and very little GIT2 (Fig. 1, D). We depleted endogenous GIT1 by specific siRNAs (short interfering RNAs) to analyze the effects on cell spreading. GIT1 silencing caused both a strong decrease of the endogenous protein (Fig. 2, A; Fig. S2), and loss of GIT signal from FAs (Fig. 2, B). It has been previously shown that GIT1 silencing by siRNA inhibits the rate of protrusion, while enhancing the stability and reducing the turnover of FAs [17]. Here, we show that GIT1 depletion inhibited COS7 cell spreading on FN (fibronectin) by negatively affecting the formation of lamellipodia and of paxillin-positive FAs at the cell edge (Fig. 2, C and Fig. S2), as previously observed after liprin-α1 silencing [11]. Quantitative analysis showed similar effects on spreading after depletion of either or both proteins (Fig. 2, D). Interestingly, no additive inhibitory effects on spreading were detected after double knockdown of liprin-α1 and GIT1 (Fig. 2, C–D), suggesting that these proteins participate into the same signaling pathway for the regulation of cell edge dynamics. In contrast to the positive effect of GIT1 in COS7 cell spreading, silencing of GIT2 causes an increase in spreading in HeLa cells, indicating that GIT2, but not GIT1, is an essential inhibitor of cell spreading and FA turnover in these cells [29]. GIT2 also inhibits cell migration, since its silencing results in a dramatic increase of transwell migration [29].

We previously found that over-expression of liprin-α1 enhances COS7 cell spreading on FN, and that this effect is prevented by depletion of the tyrosine phosphatase LAR (leukocyte common antigen-related), a binding partner of liprin-α1 [11]. Similarly, we found here that silencing of GIT1 alone or in combination with LAR knockdown prevented liprin-α1-enhanced cell spreading (Fig. 2, A,E). These data support the hypothesis that GIT1 and LAR contribute with liprin-α1 to regulate integrin-mediated spreading on extracellular matrix as part of a common signaling network.

Liprin-α1 overexpression is known to enhance the spreading of COS7 cells. We tested two different fragments of liprin-α1 to identify regions of the protein responsible for the effects on spreading: the central liprin-F3 fragment (amino acid residues 347–675), including the GIT1-binding region (Fig. S1, G) [27], and the carboxy-terminal liprin-F1F2 fragment including the three SAM (sterile alpha motif) domains (Fig. S3, A). We found that liprin-F3 was sufficient to change the morphology of the cells and to enhance spreading on FN and lamellipodia, while liprin-F1F2 had no evident effects on spreading or lamellipodia (Fig. S3, B–C).

We then tested if the direct interaction of GIT1 with liprin-α1 was necessary for the positive effects of liprin-α1 on cell spreading. We compared spreading of cells transfected with either full length liprin-α1 or liprin-ΔCC3, a deletion mutant that interacted poorly with the carboxy-terminal portion of GIT1, as detected by coimmunoprecipitation experiments (Fig. S4, A). Liprin-ΔCC3 includes the deletion of residues 615–673 of liprin-α1, a predicted coiled coil region included in the smallest fragment of liprin-α1 interacting with GIT1 [27]. Like the full length protein, also liprin-ΔCC3 remained associated to the cytoplasmic side of the plasma membrane of cells, prepared by hypotonic shock as described previously [30] (Fig. S4, B). This finding shows that the interaction with GIT1 is not needed for the localization of liprin-α1 at the cytoplasmic face of the plasma membrane of adherent cells.

The disruption of the interaction of liprin-α1 with GIT1 only mildly reduced the positive effects of liprin-α1 overexpression (Fig. S4, C), thus resulting in a more limited enhancement of spreading and F-actin-positive lamellipodia compared to the full length liprin-α1 (Fig. S4, D–E). These results suggest that, although not crucial for liprin-α1-induced spreading and F-actin reorganization at the cell edge, the association of GIT1 to liprin-α1 supports the efficiency of these processes. Therefore, the requirement of GIT1 for cell spreading is at least partially independent from its physical association to liprin-α1.

FA turnover at the cell edge is important for cell motility and spreading. Liprin-α1-induced active β1 integrin redistribution at the ventral surface of adhering cells correlates with increased spreading on FN [11]. Analysis of the distribution of FAs detected with the 9EG7 mAb specific for the activated β1 integrins (Fig. S5, A), or by antibodies for paxillin (Fig. S5, B) showed that like liprin-α1 full length, also liprin-ΔCC3 induced a decrease of the cell area occupied by FAs, and their relocalization at the cell edge. The quantification showed that liprin-ΔCC3-expressing cells had a less pronounced decrease of the total FA area (Fig. S5, C), and less evident accumulation of new FAs at the cell edge (Fig. S5, D). This was reflected by a higher fraction of liprin-ΔCC3-expressing cells with low density of FAs at the cell edge (Fig. S5, E). Therefore, although the interaction between GIT1 and liprin-α1 is not essential for the redistribution of FAs induced by liprin-α1 overexpression, it appears to affect the efficiency of this process.

### Liprin and GIT reciprocally regulate their subcellular localization

**Figure 3:**
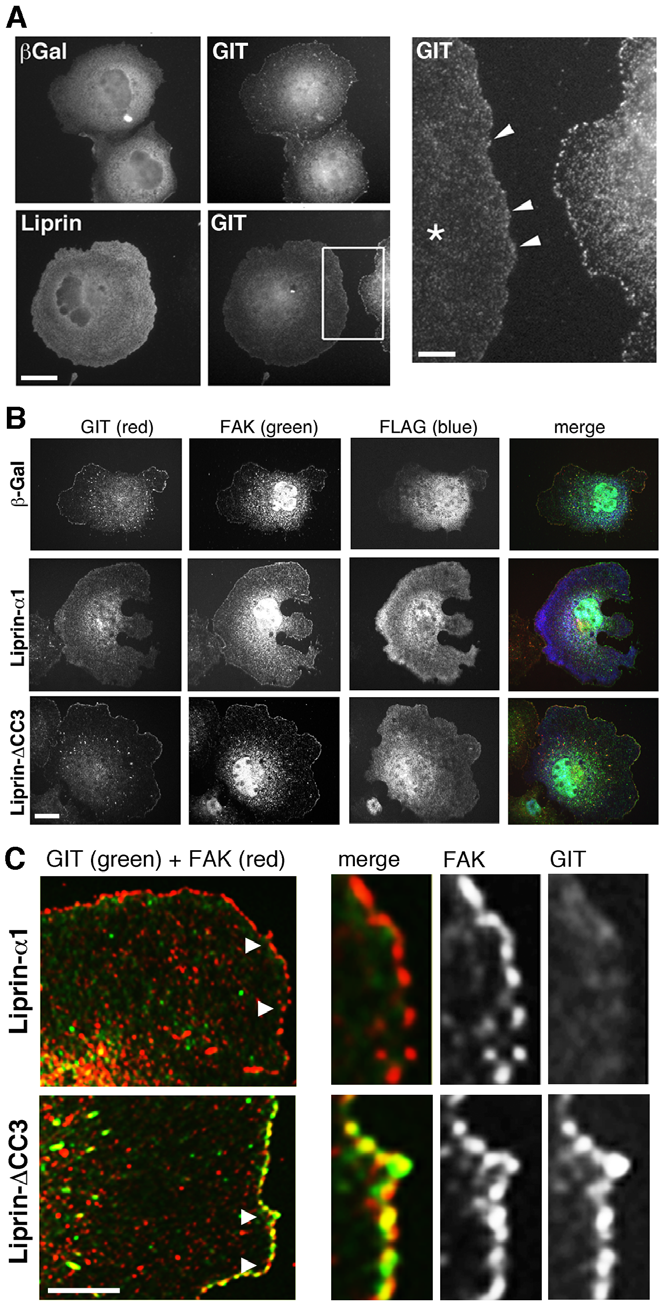
Liprin-α1 affects the subcellular localization of endogenous GIT. (A) Overexpression of liprin-α1 affects the localization of endogenous GIT at peripheral FAs. COS7 cells overexpressing either FLAG-liprin-α1 or FLAG-βgalactosidase were plated for 1 h on FN and immunostained for the transfected protein and for endogenous GIT. Scale bar, 20 µm. Right panel: four-fold enlargement of the boxed field; liprin-α1 overexpression (cell with asterisk) reduces the accumulation of GIT at newly formed FAs at the edge of transfected cells (arrowheads). Scale bar, 5 µm. (B) Cells transfected with FLAG-βgalactosidase, FLAG-liprin-α1, or FLAG-liprin-ΔCC3 were plated for 1 h on FN before fixation and staining for the transfected protein and for endogenous GIT and FAK proteins. Scale bar, 20 µm. (C) High magnification of the edge of transfected cells showing that endogenous GIT overlaps well with FAK at peripheral FAs of FLAG-liprin-ΔCC3 transfected cells, while poor overlap between endogenous GIT and FAK is seen at peripheral FAs of FLAG-liprin-α1 expressing cells. Scale bar, 10 µm. Panels on the right are 3-fold enlargements of the areas indicated by arrowheads in the corresponding images on the left.

As previously reported [4], [16], we found that endogenous GIT1 localized with paxillin to peripheral and central FAs in COS7 cells (Fig. 2, B). Intriguingly, endogenous GIT1 was relocalized following overexpression of liprin-α1 (Fig. 3, A). The localization of GIT1 was decreased both at the newly formed small FAs at the edge of spreading cells, as well as at central, mature FAs (Fig. 3, B–C). Liprin-α1 overexpression caused the specific loss of endogenous GIT from FAs, while endogenous FAK (Fig. 3, B–C) and paxillin (data not shown) remained at FAs. Also in HeLa cells, the effect of liprin-α1 overexpression was the specific removal of GIT from FAs, while the localization at FAs of paxillin and talin was not affected (Fig. S6). Interestingly, liprin-ΔCC3 expression did not affect the localization of endogenous GIT1 at peripheral FAs in COS7 cells (Fig. 3, B–C). In fact, while overexpression of the full length liprin-α1 caused a reduction of the localization of endogenous GIT1 at FAK-positive FAs, leaving a diffuse cytoplasmic signal for endogenous GIT, in cells expressing liprin-ΔCC3 GIT1 remained at peripheral FAK-positive FAs (Fig. 3, B–C). These data indicate that the direct interaction of liprin-α1 with GIT1 is required for the removal of GIT1 from FAs.

We have previously shown that GIT1 exists in an inactive state, with poor binding capacity for paxillin or liprin-α1, even when overexpressed together with βPIX in COS7 cells. On the other hand we have previously shown that different deletions within the GIT1 polypeptide induced more efficient binding of either paxillin or liprin-α1 to GIT1 [26]. Activation was detectable as the increased binding of paxillin and liprin-α1 to those deletion constructs with respect to binding to the full length GIT1. In these “activated” mutants all or part of the aminoterminal region of the GIT1 polypeptide had been removed, leaving the full carboxy-terminal portion of the protein [26]. All the data obtained by us on the putative active form of GIT1 have the limitation of being derived from the deletion of a significant part of the GIT1 polypeptide that may affect the overall structure of the protein. On the other hand, the preservation in these mutants of efficient binding to established GIT1 partners such as paxillin and liprin-α1 [8], [26], [31] is indicative of the fact that a transition between an inactive (poor binding to partners) and an active state (efficient binding to partners) may exist in the full length protein. The work by Ko et al. has shown for the first time the co-immunoprecipitation of the full length GIT1 and liprin-α1 proteins from transfected HEK293 cells [8] and from a synaptosomal fraction of adult rat brain [27]. This apparent incongruity with our model of GIT1 activation may be due to the different lysates used, and/or the different experimental conditions for immunoprecipitation used in the two laboratories. On the other hand, it can not be excluded that the interactions observed in these studies may simply reflect the less efficient binding of liprin-α1 to what we have defined as the inactive form of GIT1. Therefore, the existence of a physiologically relevant intramolecular mechanism for the activation of GIT1 at proper places and times in the cell remains an intriguing open question. To prove if this hypothesis reflects the way GIT1 is turned on in the cell, and to test whether the proposed activation occurs by an intramolecular conformational change or by proteolytic cleavage of the GIT1 polypeptide will require further experimental evidence.

**Figure 4:**
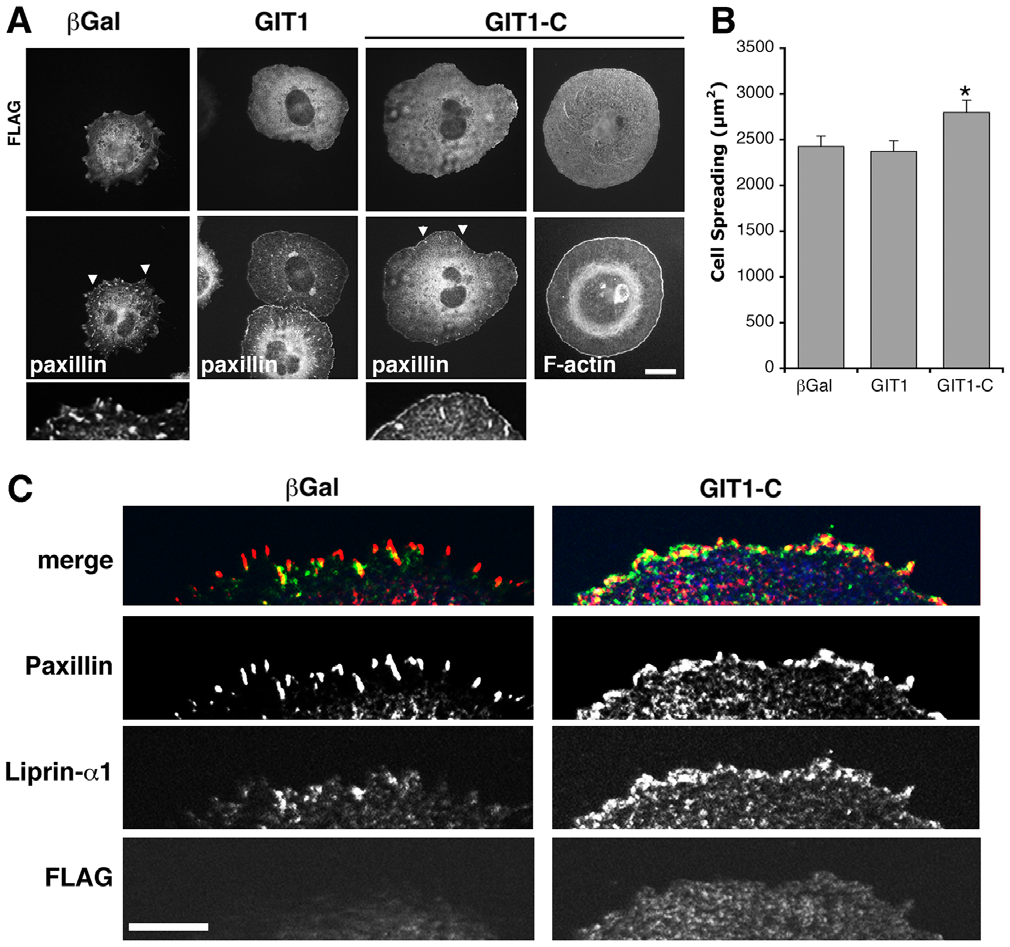
Expression of GIT1-C affects cell morphology and the distribution of endogenous liprin-α1. (A) COS7 cells transfected for one day with either FLAG-GIT1, FLAG-GIT1-C, or FLAG-βGalactosidase were re-plated for 1 h on FN. Immunofluorescence for the transfected proteins (FLAG), paxillin, and phalloidin staining for F-actin. Scale bar, 20 µm. Below, 3-fold enlargements of areas from cells stained for paxillin (arrowheads in the corresponding cells above) are shown. (B) Expression of GIT1-C induces a significant increase of cell spreading on FN. Bars are means ± SEM (n = 116–121 cells per condition); *P<0.05. (C) Cells transfected with either FLAG-βGalactosidase or FLAG-GIT1-C were used for triple immunofluorescence staining with antibodies for endogenous liprin-α1, paxillin, and transfected proteins (FLAG): endogenous liprin-α1 accumulates at the edge of GIT1-C-transfected cells. Scale bar, 5 µm.

Among the “activated” forms of GIT1, we have shown that GIT1-C (Fig. S1, H, amino acid residues 346–740) was able to specifically increase cell spreading and the reorganization of the cell edge, while overexpression of the full length protein did not show evident effects on spreading when compared to control cells (Fig. 4, A–B). Similar to what we observed after liprin-α1 overexpression, GIT1-C induced the loss of paxillin-positive FAs from the central part of the spreading cell, and the concentration of paxillin-positive small FAs at the cell edge (Fig. 4, A). To further examine the interplay between liprin-α1 and GIT1 during cell spreading, we tested the effects of the expression of the truncated active GIT1-C protein on the localization of endogenous liprin-α1 at the cell edge of spreading cells. The expression of GIT1-C, which can bind either paxillin or liprin-α1 (Fig. S1), was able to enhance the accumulation of endogenous liprin-α1 to the cell edge, where liprin partially colocalized with the paxillin-positive FAs (Fig. 4, C). The colocalization of liprin-α1 with paxillin-positive FAs was much more evident in cells transfected with GIT1-C compared to control cells.

Altogether these data indicate that liprin-α1 and activated GIT1 may reciprocally affect each other's distribution at/near the cell edge during active integrin-mediated cell motility. Liprin-α1 overexpression decreases the localization of endogenous GIT1 at both peripheral, and mature central FAs in spreading cells. On the other hand, the expression of an active form of GIT1 induces the concentration of endogenous liprin-α1 at the edge of spreading cells. We hypothesize that this interplay between liprin-α1 and GIT1 may be necessary for the dynamic reorganization of the adhesive sites and the cytoskeleton of spreading cells, thus possibly promoting the turnover of FAs. The changes in the organization of the cell edge observed when the levels of either protein were altered, and the effects on cell spreading are indications in support of the proposed functional interaction between liprin-α1 and GIT1.

### GIT1 is required for liprin-α1-enhanced haptotactic COS7 cell migration

**Figure 5:**
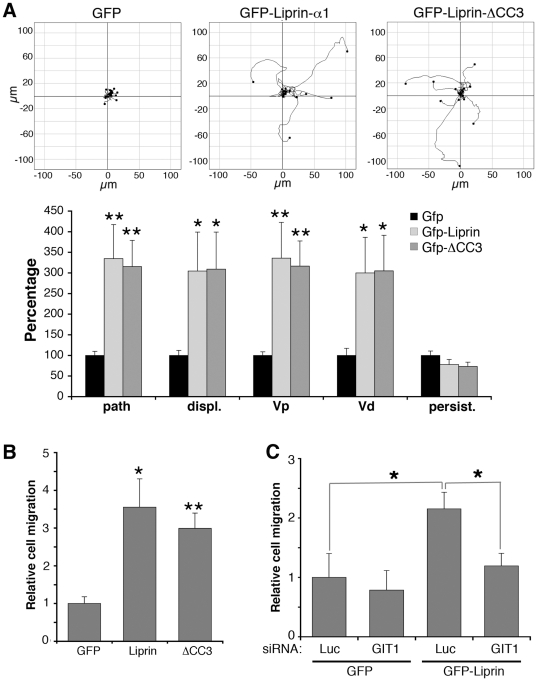
GIT1 is required for liprin-α1-enhanced COS7 cell migration. (A) Transfected cells were replated on 10 µg/ml FN for 50 min to allow spreading, and then monitored for motility for 2.5 h by taking one frame every 5 min. The upper panels show cell tracks from cells transfected with the indicated constructs. The lower panel shows the quantification (mean values ±SEM) of different parameters of random migration including cell tracks (path), Euclidean distance (displ.), path rate (Vp), Euclidean rate (Vd) and persistence of migration (persist = path/displ.). N = 18–20 cells per experimental condition; *P<0.05. (B) Transwell migration assays with cells transfected with GFP, GFP-liprin-α1, or GFP-liprin-ΔCC3. Bars are normalized means ± SEM (n = 4); *P<0.05; **P<0.01. (C) Transwell migration assays with cells cotransfected with the indicated combinations of siRNAs and plasmids. Bars are normalized means ± SEM (n = 4); *P<0.05.

We have used a random migration assay to analyze the role of the liprin-α1/GIT1 complex in a different motility assay. COS7 cells were poorly motile when tested in a random migration assay on FN, while they became active after overexpression of liprin-α1 (Fig. S7). No differences were evident between cells expressing either GFP-liprin-α1 or the GIT1 binding-deficient mutant GFP-liprin-ΔCC3 (Fig. 5, A). Therefore, the interaction between liprin-α1 and GIT1 is not essential to regulate the random motility of COS7 cells. Similar results were obtained by using a haptotactic transwell migration assay, in which COS7 cell migration towards a FN-coated substrate was strongly enhanced by liprin-α1 overexpression, but also by the expression of the GIT1 binding-deficient mutant liprin-ΔCC3 (Fig. 5, B). On the other hand, we found that endogenous GIT1 was required for liprin-α1-enhanced migration (Fig. 5, C). Previous findings have shown that overexpression of GIT1 enhanced haptotactic COS7 cell migration [4] and CHO-K1 cell migration on FN [16], while GIT1 depletion prevented formyl-Met-Leu-Phe peptide-enhanced chemotaxis of rat basophilic leukaemia RBL cells [32]. Although silencing the endogenous GIT1 protein did not significantly affect basal cell migration, it prevented the potentiation of transwell migration induced by liprin-α1 overexpression (Fig. 5, C). Altogether these data indicate that the function of GIT1 is important for liprin-α1-mediated migration, although a direct interaction between the two proteins is not necessary.

### Conclusions

During cell spreading and migration on extracellular matrix, continuous reorganization of FAs and actin dynamics at the cell front are necessary for effective protrusion [33]. Given the implication of GIT1 and its partners paxillin and liprin-α1 in the regulation of cell edge dynamics, the interaction of GIT1 with either partner may represent two distinct functional states of GIT1 during cell motility. This is supported by our biochemical data suggesting that binding of liprin-α1 competes for binding of paxillin to the carboxy-terminal portion of GIT1 (Fig. 1, A). Moreover, the hypothesis is also supported by the functional analysis showing that the localization of endogenous GIT1 and liprin-α1 is reciprocally influenced by the other partner with respect to the paxillin- and FAK-positive FAs at the dynamic edge of spreading cells (Figs. 3, 4). The requirement of distinct complexes including different combinations of the partners may be expected, if we consider the complexity of the scaffold proteins involved and of the cellular processes underlying cell motility.

The carboxy-terminal paxillin binding region of GIT1 is critical for GIT1 function, since mutants of GIT1 lacking this region fail to regulate cell migration and protrusion [34]. In particular, phosphorylation of serine 709 within the paxillin binding region is necessary for the effects of GIT1 on protrusions and to increase its binding to paxillin, which could target GIT1 to the leading edge of cells [34]. Therefore, one could envisage that competitive binding of liprin-α1 to GIT1 displaces GIT1 from paxillin. As a consequence, paxillin would remain at FAs while GIT1 would be recycled to the cytoplasm. Accordingly, we found that overexpression of liprin-α1, but not of the GIT1-deficient liprin-ΔCC3 mutant, was able to dramatically displace endogenous GIT1 from FAs (Fig. 3), while leaving paxillin at these sites (Fig. S5).

Paxillin plays a positive role in FA formation/turnover: it is one of the earliest proteins found associated to newly formed FAs at the protruding cell edge [35]. On the other hand, paxillin appears to regulate also the disassembly of FAs, since lack of paxillin leads to the formation of more stable adhesions [36]. Our previous work has shown that the ability of different paxillin-binding GIT1 deletion mutants to inhibit cell spreading correlated with their inhibitory effects on the localization of paxillin at vinculin-positive FAs. On the other hand, the increased ability of GIT1-C to promote spreading was accompanied by the enhanced localization of paxillin at peripheral FAs [26]. Altogether, our findings support the hypothesis that GIT1, once activated, may act as a transporter for paxillin within the cell, while liprin-α1 negatively affects the accumulation of endogenous GIT1 at FAs without affecting the localization of paxillin at these sites. We have been able to show the interaction between GIT1 and its two partners only by using GIT1 deletion mutants corresponding to an “activated” form of GIT1. It could be envisaged that endogenous GIT1 is locally activated in the cell by so far unknown mechanisms, which would allow then the interaction of GIT1 with the distinct partners during different phases of cell edge protrusion.

Our findings show that GIT1 and its partner liprin-α1 are both required for the reorganization of the cell edge during spreading on extracellular matrix, since depletion of either protein causes a similar inhibition of cell spreading on FN. The inhibitory effects observed on spreading are not additive after silencing both proteins, while the positive effects of liprin-α1 overexpression on spreading and migration can be prevented by the downregulation of endogenous GIT1. These observations support the hypothesis that the two proteins cooperate in the same pathway during COS7 cell motility.

In conclusion, the data presented in this study lead us to propose a model in which the alternative binding of liprin-α1 or paxillin to GIT1 plays distinct roles in different phases of the protrusive activity of the cell. It will be interesting to test in future studies the hypothesis that GIT1 and liprin-α1 play distinct, possibly sequential roles during protrusion by specifically addressing the role of each of the two scaffolds in the sequence of events leading to cell edge protrusion.

## Materials and Methods

### Antibodies

The antibodies used in this study were as follows: monoclonal antibodies (mAb) anti-FLAG M5 and M2, anti-talin, and anti-tubulin (Sigma-Aldrich, Saint Louis, MO); anti-HA 12CA5, anti-Myc 9E10 (Primm Biotech, Milano, Italy); anti-paxillin, anti-GIT/PKL, anti-LAR recognizing the 150 kDa form, and mAb 9EG7 recognizing activated human β1 integrins [37] (BD Transduction Laboratories, San Jose, CA). Polyclonal antibodies (pAb) anti-FLAG and anti-actin (Sigma-Aldrich); anti-FAK and anti-GIT1 (Santa Cruz Biotechnology, Santa Cruz, CA); pAbs for βPIX, GIT1, GIT2, and liprin-α1 were described previously [11], [29], [38]–[39]. FITC- and TRITC-conjugated phalloidin were from Sigma-Aldrich.

### DNA constructs and siRNAs

Several constructs derived from GIT1 (Fig. S1, H) were cloned into the pFLAG-CMV2 vector (Eastman Kodak, Inc. Rochester, NY) or into the pBK-haemagglutinin (HA) vector derived from pBK-CMV (Stratagene, Santa Clara, CA). Full length, deletion mutants, and fragments of liprin-α1 were cloned into the pFLAG-CMV2 (Kodak) and the pcDNA3.1(–)/ Myc-His vectors (Invitrogen, Paisley, Scotland, UK). The cDNA for GIT1-C2 was cloned into the pQE60ZZ vector, derived from pQE60 (Qiagen, Hilden, Germany) including the sequence coding for a “ZZ” tag (two consecutive IgG binding domains of protein A), to obtain the pQE60ZZ-GIT1-C2 plasmid. SiRNA for liprin-α1 and LAR were previously described [11]. The GIT1a and GIT1b siRNAs (Invitrogen) targeted the sequences 5′-GCCTGGATGGAGACCTAGA-3′ and 5′-AGCCAACCCCCAAGACAAATT -3′ of human green monkey GIT1, respectively.

### Cell culture and transfection

COS7 and HeLa cells (from the American Type Culture Collection, Teddington, UK) were grown in Dulbecco's modified Eagle's medium (Cambrex Bio Science Verviers SPRL, Charles City, IA) with 10% serum. Cells transfected with Lipofectamine 2000TM (Invitrogen) or Fugene (Roche, Manheim, Germany) and 2–3 µg of plasmids, or siRNAs (50–100 nM) were used after 1–2 days, respectively.

### Immunoprecipitation and immunoblotting

Cells were lysed with 0.5–1% Triton X-100, 20 mM Tris-HCl pH 7.5, 150 mM NaCl, 1 mM sodium orthovanadate, 10 mM sodium fluoride, and protease inhibitors. Aliquots of 200–1,000 µg of each lysate were incubated with the indicated antibodies pre-bound to Protein A-Sepharose beads (Amersham, Little Chalfont, UK). Immunoprecipitation of endogenous paxillin was performed by conjugating protein A Sepharose beads to 2 µl of rabbit anti-mouse Ig (Sigma-Aldrich) and 1 µg of anti-paxillin mAb (BD Biosciences Transduction Laboratories). For immunoblotting primary antibodies were visualized by ECL or ^125^I-anti-mouse Ig or Protein A (Amersham).

### Mass spectroscopy analysis

BL21 bacteria transformed with pQE60ZZ-GIT1-C2 were used to express the ZZ-GIT1-C2 fusion protein upon induction with IPTG. The ZZ-GIT1-C2 fusion protein includes a carboxy-terminal GIT1 fragment linked to two consecutive IgG binding domains of protein A. Bacteria were lysed and the fusion protein was purified on IgG Sepharose 6 beads (Amersham). For the purification of ZZ-GIT1-C2-binding proteins, all procedures were carried at 0–4°C. 45 mg of protein from E12-E13 chick brain lysate (lysis buffer: 1% Triton X-100, 20 mM Tris-HCl pH 7.5, 150 mM NaCl, 0.1 mM DTT, 10 mM sodium orthovanadate, 1 mM sodium fluoride, anti-protease mixture) were incubated with ZZ-GIT1-C2 preadsorbed to 75 µl of IgG-beads. After incubation for 1.5 h with rotation, beads were washed, transferred to a column, further washed thoroughly, and eluted twice with 0.5 M acetic acid (pH 3.4). Control samples included IgG Sepharose beads incubated with brain lysate (in the absence of ZZ-GIT1-C2 protein), and IgG Sepharose beads coated with ZZ-GIT1-C2 protein (in the absence of brain lysate). One fourth of each eluate and of the beads left after elution with acetic acid were analyzed by SDS-PAGE on 6% acrylamide gels.

For protein identification, bands of interest were excised from silver-stained SDS–PAGE gels, reduced, alkylated and digested overnight with bovine trypsin as described elsewhere [40]. One µl of the supernatant of the digestion was used for MALDI-time of flight mass spectrometer (TOF MS) analysis using the dried droplet technique and α-cyano-4-hydroxycinnamic acid as matrix. All analyses were performed using a Voyager-DE STR (Applied Biosystems) TOF MS operated in the delayed extraction mode. Peptides were measured in the mass range from 750 to 4,000 Da; all spectra were internally calibrated and processed via the Data Explorer software. Proteins were unambiguously identified by searching a comprehensive non-redundant protein database using the program ProFound [41].

### Cell spreading assays

Cells were trypsinized 1–2 days after transfection. 25,000–30,000 cells were plated on 13 mm diameter coverslips coated with 10 µg/ml FN. Cells were fixed after 1 h and processed for immunofluorescence. Images were analyzed with ImageJ (Bethesda, MD). Significance was set at P<0.05, by the Student's t test.

### Haptotactic and random migration assay

Transfected cells were incubated overnight in serum-free medium, trypsinized, and 30,000 cells/transwell were seeded in serum-free medium (8 µm pore PET membrane, Millipore, Billerica, MA). The lower side of the chambers were coated with 20 µg/ml of FN, and filled with DMEM without serum. After 8 h at 37°C non-migrating cells were removed from the upper chamber, and cells on the lower side were fixed with 3% paraformaldehyde and detected by immunofluorescence. For quantification, GFP positive cells were counted from 6 representative fields per well (20× lens). Data were collected from 4 independent experiments, each in duplicate. Values of migrated cells were normalized with respect to the percentage of transfected cells (between 30 and 60% transfection efficiency). Random migration was performed and quantified as previously described [24].

### Morphological analysis

Ventral plasma membranes were prepared by hypotonic shock of COS7 cells as previously described [11], [30]. Cells and ventral plasma membranes were incubated with the indicated antibodies after fixation. F-actin was revealed by FITC- or TRITC-conjugated phalloidin. Cells were observed with Axiophot or Axiovert microscopes (Zeiss, Oberkochen, Germany), or confocal microscopes (PerkinElmer, Waltham, MA and Leica Microsystems GmbH, Wetzlar, Germany). For immunofluorescence, images within the same panels were acquired and treated identically for comparisons. Images were processed using Photoshop (Adobe) and analyzed for cell spreading and FA area with ImageJ as described before [11]. Data in the bar graphs are expressed as mean ± SEM from at least 2–3 repetitions in which 70–150 cells per experimental conditions were analyzed. Random migration was analyzed as previously described [24]. P values were calculated by the Stutent's t-test (two-tailed distribution, two-sample unequal variance).

## Supporting Information

Figure S1 **Characterization of the binding of liprin-α to GIT1-derived polypeptides.** (A–B) Interaction of liprin-α with GIT1-C2. Lane 1, control IgG-beads coated with the ZZ-GIT1-C2 fusion protein; lane 2, IgG-beads coupled to the ZZ-GIT1-C2 fusion protein and incubated with 45 mg of E15 chicken brain lysate; lane 3, control IgG-beads incubated with 45 mg of E15 chicken brain lysate without the ZZ-GIT1-C2 fusion protein. After washing, in lane 2, a band of about 160 kDa was specifically eluted with respect to the control lanes 1 and 3. Analysis by mass spectroscopy identified the avian 160 kDa polypeptide (asterisk) as a close homologue of human liprin-α2. (B) Aminoacid sequence of human liprin-α2. In grey are indicated the peptides corresponding to the highly homologous avian peptides identified by mass spectroscopy of the 160 kDa eluted from the IgG-beads coupled to the ZZ-GIT1-C2 fusion protein and incubated with E15 chicken brain lysate (see lane 2 of panel A). (C–E) Liprin-α1 and paxillin interact with GIT1 fragments in cells. Immunoprecipitations (IP) from lysates of COS7 cells transfected with the indicated FLAG-GIT1-derived constructs alone or in combination with Myc-liprin-F3. After immunoprecipitation of either liprin-F3 (anti-Myc Ab) or endogenous paxillin, filters with immunoprecipitates and lysates were probed by immunoblotting for liprin-F3, GIT1 constructs, or endogenous paxillin. The data in (C–E) show that the liprin fragment F3 interacts with GIT1-C2, but not with shorter fragments of the carboxyterminus of GIT1. On the other hand, paxillin is also able to bind weakly to the shorter carboxyterminal GIT1(512–740) fragment. (F) Lysates (300 µg) from cells transfected with either FLAG-GIT1-C2 or FLAG-GIT1-C were immunoprecipitated with antibodies for endogenous paxillin (left) or endogenous liprin-α1 (center). Immunoprecipitates and lysates were then blotted with anti-FLAG antibodies to identify the transfected FLAG-GIT1 constructs. The results show that both endogenous paxillin and endogenous liprin-α bind the carboxyterminal GIT1 constructs. Lysates (50 µg each) are shown to the right. (G) Scheme of the liprin-α1 and liprin-F3 constructs. (H) Summary of some of the constructs tested: a more extended carboxy-terminal portion of GIT1 is required for binding to liprin-α compared to paxillin. ArfGAP, ArfGAP domain; Ank's, ankyrin repeats; SHD, Spa2 homology domain; CC coiled coil region; PBD, paxillin binding domain.
(TIF)

Figure S2 **Silencing of GIT1 with either of two different siRNAs inhibits cell spreading.** Left: equal amounts of protein lysates from COS7 cells transfected with the indicated siRNA were immunoblotted for GIT proteins (upper filter) or tubulin (lower filter). Molecular weight markers are indicated on the left. Right: quantification of the effects of control and GIT1-specific siRNAs on spreading of cells plated 1 h on FN (n = 70–150 cells per condition from 2–3 experiments). *P<0.05; **P<0.01.
(TIF)

Figure S3 **The GIT1-binding liprin-F3 fragment is sufficient to enhance cell spreading.** (A) FLAG-tagged liprin-α1 constructs used in this study. (B) Transfected COS7 cells were plated for 1 h on FN. Scale bar, 20 µm. (C) Quantification of spreading after 1 h on FN. Bars are mean values ± SEM (n = 50 cells; **P<0.01).
(TIF)

Figure S4 **Effects of liprin-ΔCC3 expression on spreading.** (A) Lysates from cells transfected with GIT1-C2, GIT1-C2 and liprin-α1, or GIT1-C2 and liprin-ΔCC3 (schemes under the blots) were immunoprecipitated (IP) with anti-liprin-α1 antibodies. Filters were analyzed by immunoblotting for the indicated antigens. (B) Immunostaining for liprin of ventral plasma membranes prepared as described in the Methods, starting from cells transfected with either full length liprin-α1 or liprin-ΔCC3. Scale bar, 20 µm. (C) Cells transfected with βgalactosidase, liprin-α1, or liprin-ΔCC3 were plated 1 h on FN and stained for the transfected protein (left) and F-actin (right). (D) Quantification of spreading in cells treated as described in (C). Bars are mean values ± SEM (n = 150 cells from 3 experiments). (E) Cells transfected with the indicated constructs and plated 1 h on FN were fixed and evaluated for the presence of lamellipodia, measured as the percentage of F-actin-positive cell perimeter. Bars are means ± SEM (n = 20 cells from 2 experiments). *P<0.05; **P<0.01.
(TIF)

Figure S5 **Liprin-α1 affects the distribution of FAs and activated integrin receptors at the cell edge in a GIT1-independent way.** (A) COS7 cells plated for 1 h on FN, and stained with the 9EG7 mAb specific for activated β1 integrins. Scale bar, 20 µm. (B) Distribution of paxillin-positive peripheral FAs at the edge of cells transfected with GFP, GFP-Liprin-α1, or GFP-Liprin-ΔCC3, and plated for 1 h on FN. Scale bar, 10 µm. (C–D) Quantification of active β1 integrin-positive FAs from transfected cells as those shown in (A): (C) fraction of projected cell area occupied by active β1-integrin-positive FAs; (D): percentage of FA area at the cell edge. Bars are means ± SEM (n = 24 cells per condition). *P<0.05; **P<0.01. (E) Percentage of spreading cells with either high (grey) or low (dark grey) FA density at the edge (n = 26 fields from 13 cells per condition; *P<0.001 by the χ2 test).
(TIF)

Figure S6 **Distribution of FA proteins in HeLa cells overexpressing liprin-α1.** HeLa cells overexpressing either FLAG-liprin-α1 or FLAG-βgalactosidase were plated for 1 h on FN and immunostained for the transfected protein and for the indicated endogenous proteins. While endogenous GIT was displaced from peripheral FAs in cells overexpressing liprin-α1, the localization at FAs of other endogenous components was not evidently affected. Asterisks indicate transfected cells. Scale bar, 20 µm.
(TIF)

Figure S7 **Effects of liprin-α1 overexpression on COS7 cell motility.** COS7 cells transfected with GFP or GFP-Liprin-α1 were plated 50 min on 10 µg/ml FN before time-lapse analysis at the indicated time points. Scale bar, 10 µm.
(TIF)
