# Supplementary figures and images for: Biochemical and Functional Characterization of the Interaction between Liprin-α1 and GIT1: Implications for the Regulation of Cell Motility
Source: PLoS One. 2011 Jun 13;6(6):e20757. doi: 10.1371/journal.pone.0020757 (PMC3113849; doi:10.1371/journal.pone.0020757)

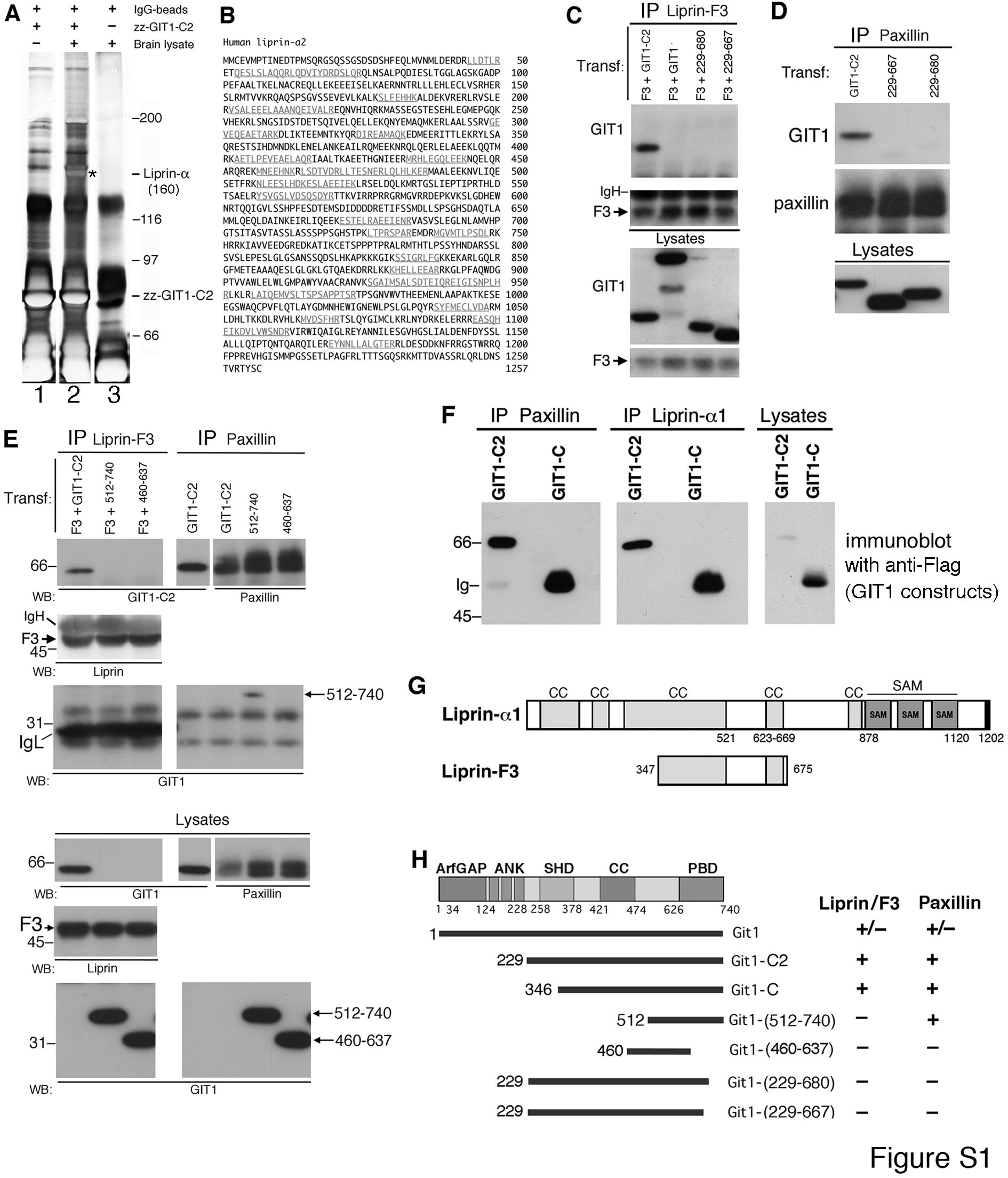

Supplement: Figure S1 — Characterization of the binding of liprin-α to GIT1-derived polypeptides. (A–B) Interaction of liprin-α with GIT1-C2. Lane 1, control IgG-beads coated with the ZZ-GIT1-C2 fusion protein; lane 2, IgG-beads coupled to the ZZ-GIT1-C2 fusion protein and incubated with 45 mg of E15 chicken brain lysate; lane 3, control IgG-beads incubated with 45 mg of E15 chicken brain lysate without the ZZ-GIT1-C2 fusion protein. After washing, in lane 2, a band of about 160 kDa was specifically eluted with respect to the control lanes 1 and 3. Analysis by mass spectroscopy identified the avian 160 kDa polypeptide (asterisk) as a close homologue of human liprin-α2. (B) Aminoacid sequence of human liprin-α2. In grey are indicated the peptides corresponding to the highly homologous avian peptides identified by mass spectroscopy of the 160 kDa eluted from the IgG-beads coupled to the ZZ-GIT1-C2 fusion protein and incubated with E15 chicken brain lysate (see lane 2 of panel A). (C–E) Liprin-α1 and paxillin interact with GIT1 fragments in cells. Immunoprecipitations (IP) from lysates of COS7 cells transfected with the indicated FLAG-GIT1-derived constructs alone or in combination with Myc-liprin-F3. After immunoprecipitation of either liprin-F3 (anti-Myc Ab) or endogenous paxillin, filters with immunoprecipitates and lysates were probed by immunoblotting for liprin-F3, GIT1 constructs, or endogenous paxillin. The data in (C–E) show that the liprin fragment F3 interacts with GIT1-C2, but not with shorter fragments of the carboxyterminus of GIT1. On the other hand, paxillin is also able to bind weakly to the shorter carboxyterminal GIT1(512–740) fragment. (F) Lysates (300 µg) from cells transfected with either FLAG-GIT1-C2 or FLAG-GIT1-C were immunoprecipitated with antibodies for endogenous paxillin (left) or endogenous liprin-α1 (center). Immunoprecipitates and lysates were then blotted with anti-FLAG antibodies to identify the transfected FLAG-GIT1 constructs. The results show that both en [file pone.0020757.s001.tif]

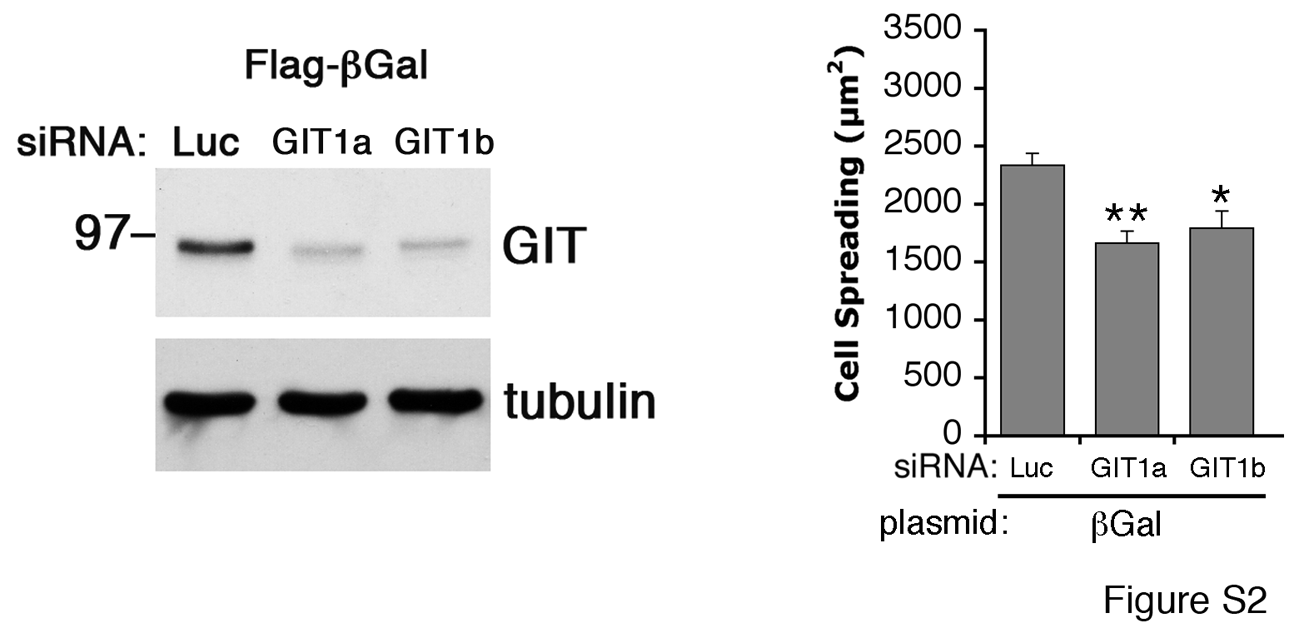

Supplement: Figure S2 — Silencing of GIT1 with either of two different siRNAs inhibits cell spreading. Left: equal amounts of protein lysates from COS7 cells transfected with the indicated siRNA were immunoblotted for GIT proteins (upper filter) or tubulin (lower filter). Molecular weight markers are indicated on the left. Right: quantification of the effects of control and GIT1-specific siRNAs on spreading of cells plated 1 h on FN (n = 70–150 cells per condition from 2–3 experiments). *P<0.05; **P<0.01. (TIF) [file pone.0020757.s002.tif]

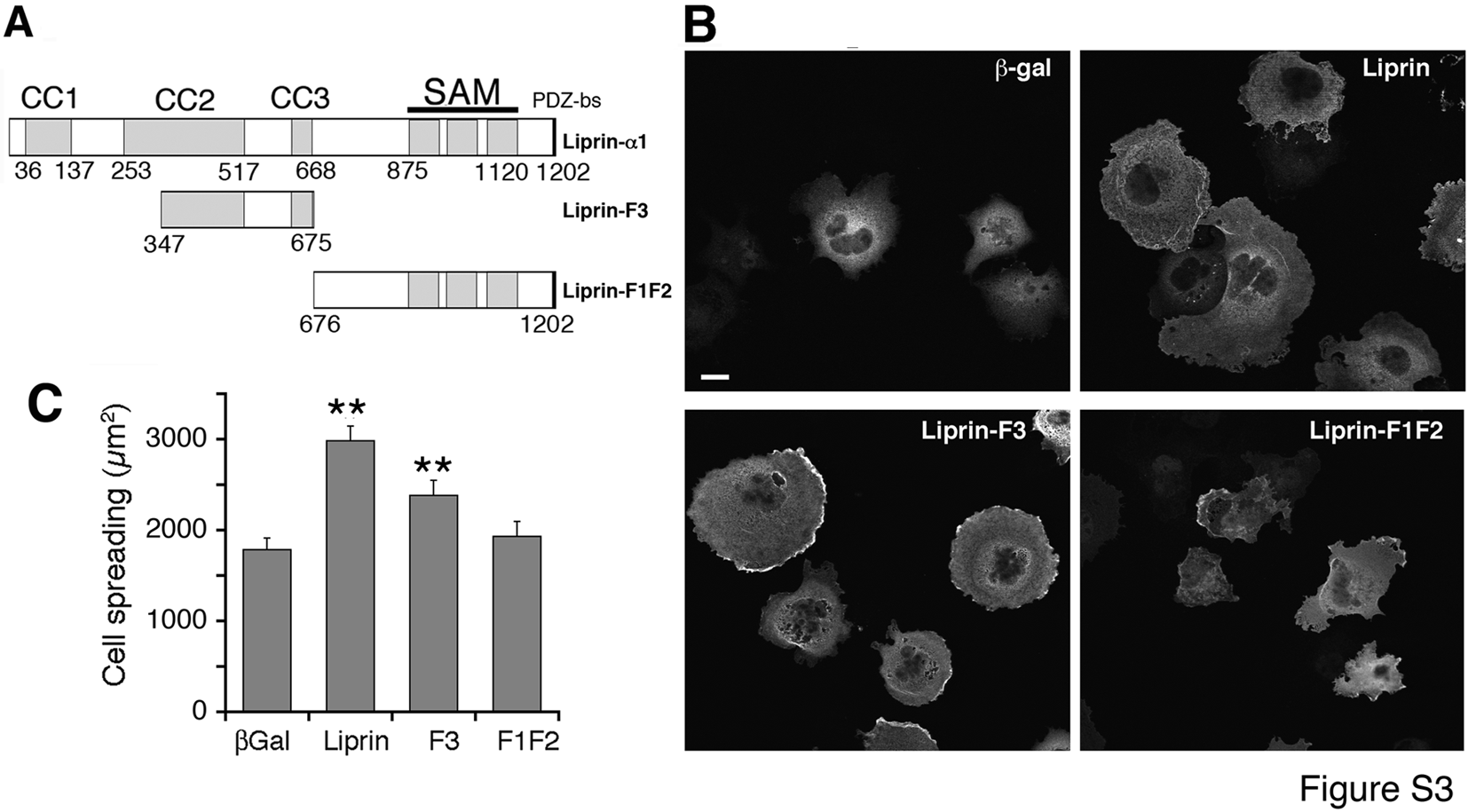

Supplement: Figure S3 — The GIT1-binding liprin-F3 fragment is sufficient to enhance cell spreading. (A) FLAG-tagged liprin-α1 constructs used in this study. (B) Transfected COS7 cells were plated for 1 h on FN. Scale bar, 20 µm. (C) Quantification of spreading after 1 h on FN. Bars are mean values ± SEM (n = 50 cells; **P<0.01). (TIF) [file pone.0020757.s003.tif]

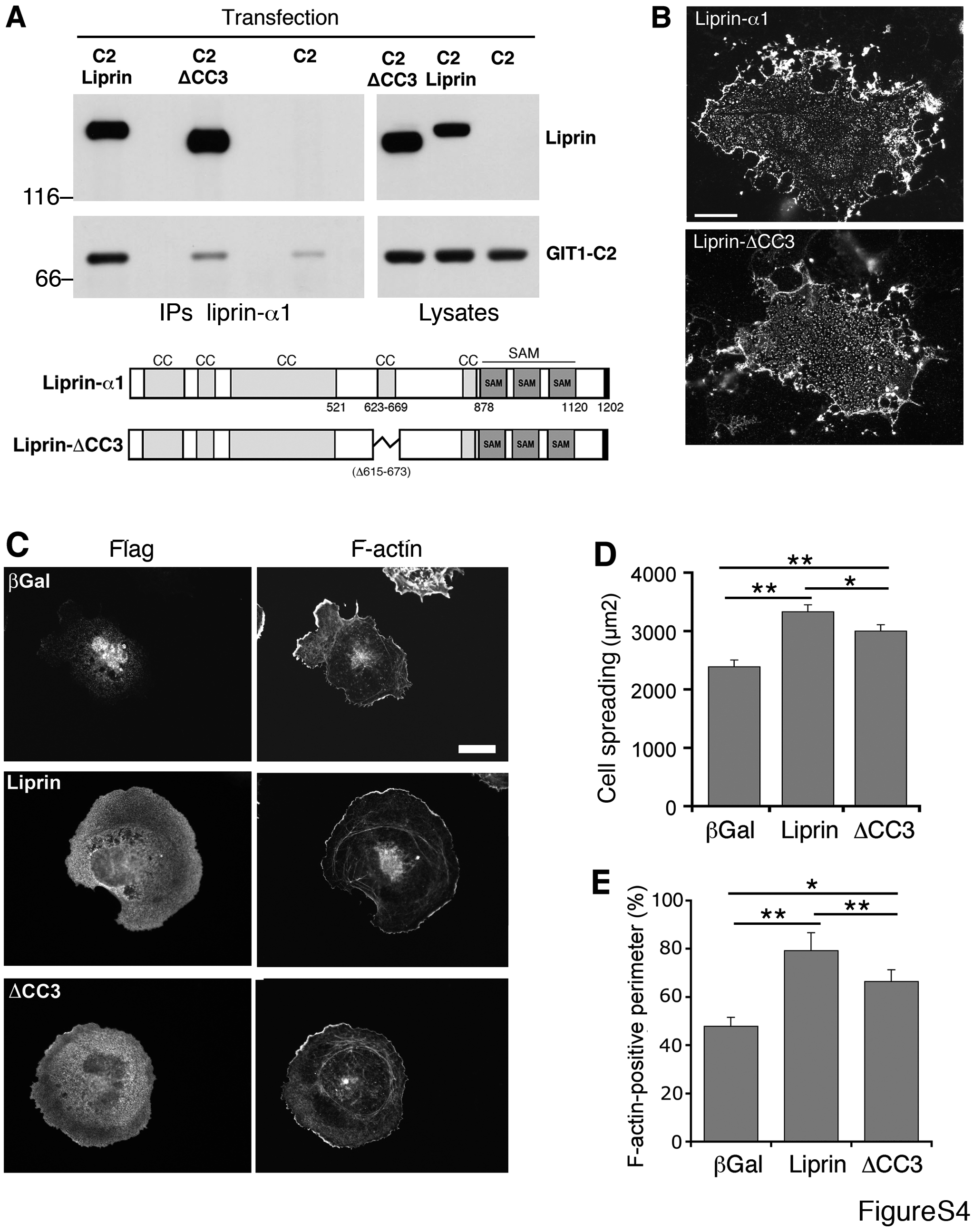

Supplement: Figure S4 — Effects of liprin-ΔCC3 expression on spreading. (A) Lysates from cells transfected with GIT1-C2, GIT1-C2 and liprin-α1, or GIT1-C2 and liprin-ΔCC3 (schemes under the blots) were immunoprecipitated (IP) with anti-liprin-α1 antibodies. Filters were analyzed by immunoblotting for the indicated antigens. (B) Immunostaining for liprin of ventral plasma membranes prepared as described in the Methods, starting from cells transfected with either full length liprin-α1 or liprin-ΔCC3. Scale bar, 20 µm. (C) Cells transfected with βgalactosidase, liprin-α1, or liprin-ΔCC3 were plated 1 h on FN and stained for the transfected protein (left) and F-actin (right). (D) Quantification of spreading in cells treated as described in (C). Bars are mean values ± SEM (n = 150 cells from 3 experiments). (E) Cells transfected with the indicated constructs and plated 1 h on FN were fixed and evaluated for the presence of lamellipodia, measured as the percentage of F-actin-positive cell perimeter. Bars are means ± SEM (n = 20 cells from 2 experiments). *P<0.05; **P<0.01. (TIF) [file pone.0020757.s004.tif]

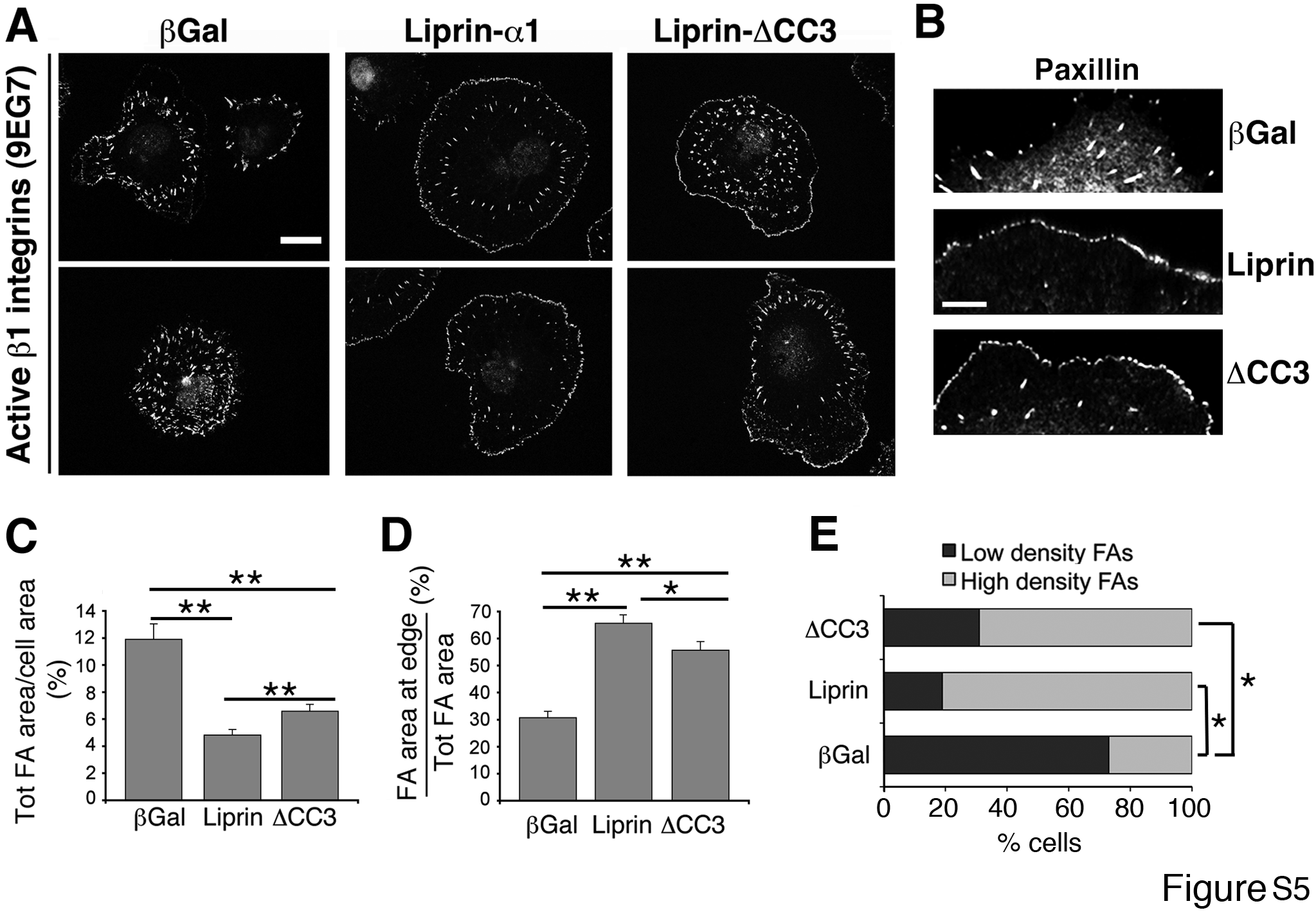

Supplement: Figure S5 — Liprin-α1 affects the distribution of FAs and activated integrin receptors at the cell edge in a GIT1-independent way. (A) COS7 cells plated for 1 h on FN, and stained with the 9EG7 mAb specific for activated β1 integrins. Scale bar, 20 µm. (B) Distribution of paxillin-positive peripheral FAs at the edge of cells transfected with GFP, GFP-Liprin-α1, or GFP-Liprin-ΔCC3, and plated for 1 h on FN. Scale bar, 10 µm. (C–D) Quantification of active β1 integrin-positive FAs from transfected cells as those shown in (A): (C) fraction of projected cell area occupied by active β1-integrin-positive FAs; (D): percentage of FA area at the cell edge. Bars are means ± SEM (n = 24 cells per condition). *P<0.05; **P<0.01. (E) Percentage of spreading cells with either high (grey) or low (dark grey) FA density at the edge (n = 26 fields from 13 cells per condition; *P<0.001 by the χ2 test). (TIF) [file pone.0020757.s005.tif]

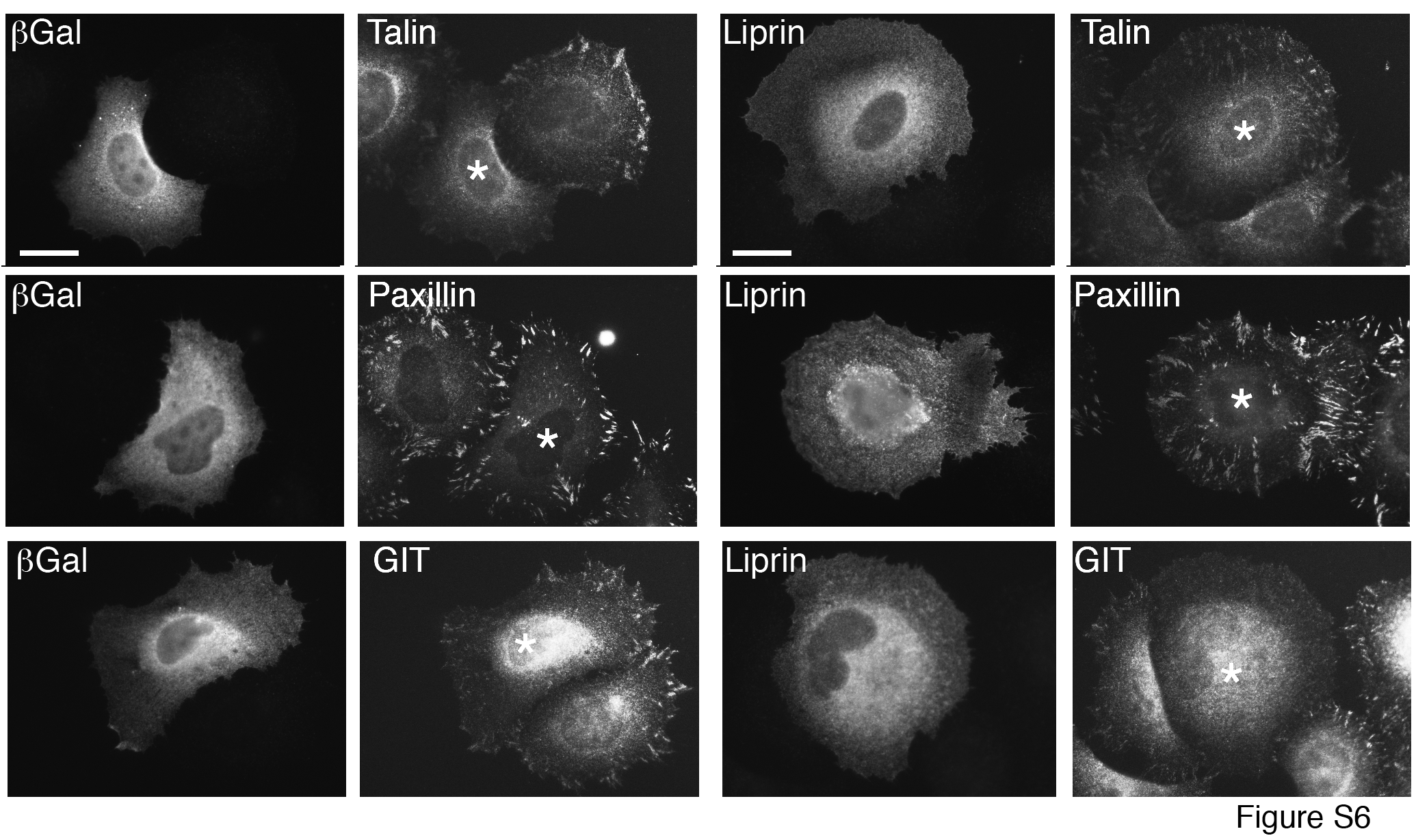

Supplement: Figure S6 — Distribution of FA proteins in HeLa cells overexpressing liprin-α1. HeLa cells overexpressing either FLAG-liprin-α1 or FLAG-βgalactosidase were plated for 1 h on FN and immunostained for the transfected protein and for the indicated endogenous proteins. While endogenous GIT was displaced from peripheral FAs in cells overexpressing liprin-α1, the localization at FAs of other endogenous components was not evidently affected. Asterisks indicate transfected cells. Scale bar, 20 µm. (TIF) [file pone.0020757.s006.tif]

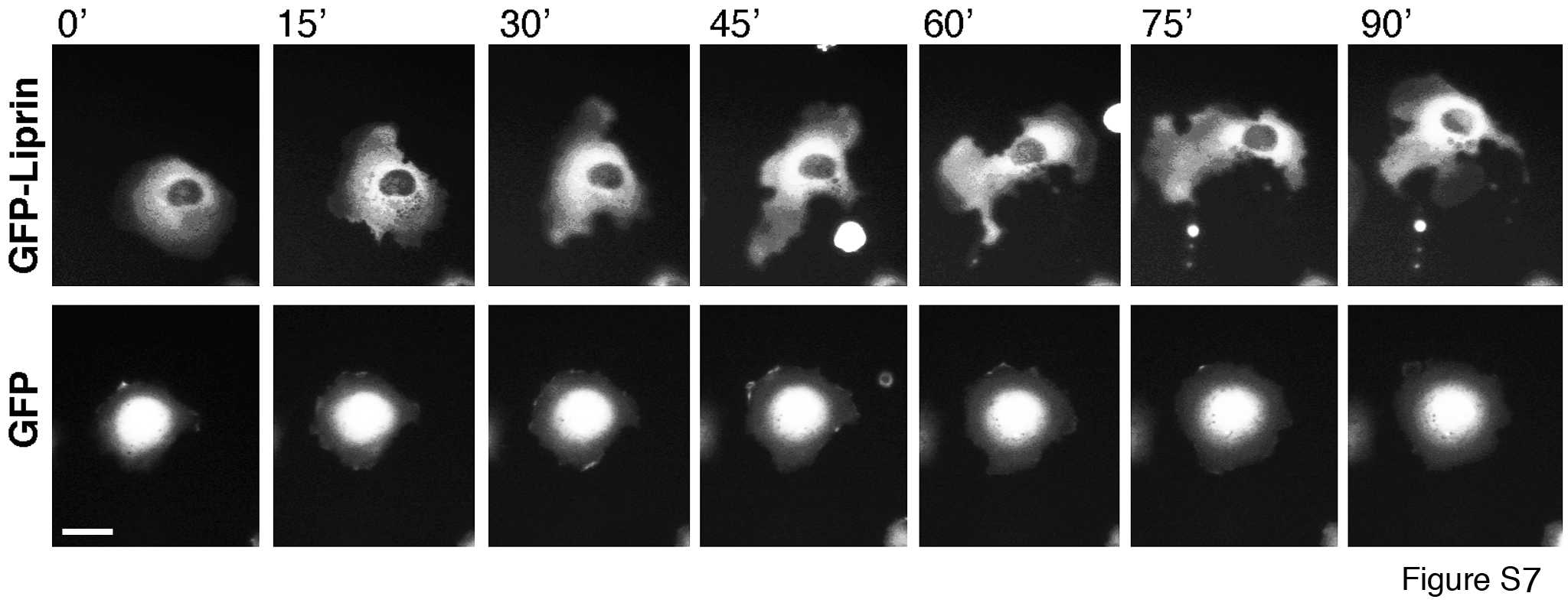

Supplement: Figure S7 — Effects of liprin-α1 overexpression on COS7 cell motility. COS7 cells transfected with GFP or GFP-Liprin-α1 were plated 50 min on 10 µg/ml FN before time-lapse analysis at the indicated time points. Scale bar, 10 µm. (TIF) [file pone.0020757.s007.tif]
